# Pathway-driven therapeutic stratification in pancreatic ductal adenocarcinoma

**DOI:** 10.1016/j.tranon.2026.102920

**Published:** 2026-07-16

**Authors:** Eduardo Chuluyan, Daniel Grasso, Analia Pasqua, Carla Remolins, Andrea Paes de Lima, Kevin Matamoros, Diego Guerrieri, Gustavo Kohan, Florencia Gottardo, Martin Ledesma, Juan Garona, Carlos Davio, Agustin Yaneff, Maria Noe Garcia, Daniela Papademetrio, Maria Eugenia Pasqualini, Luis Sarotto, Jonathan Garnier, Brice Chanez, Oscar Mazza, Nicolas Fraunhoffer, Nelson Dusetti, Juan Iovanna

**Affiliations:** a Programa Franco-argentino de Estudio del Cáncer de Páncreas, Buenos Aires, Argentina; b Centro de Estudios Farmacológicos y Botánicos (CEFYBO), Facultad de Medicina, Universidad de Buenos Aires-CONICET, Argentina; c Departamento de Microbiología, Parasitología e Inmunología, Facultad de Medicina, Universidad de Buenos Aires, Buenos Aires, Argentina; d Joint Lab INSERM-CONICET-UBA-UNAJ-HEC, Argentina; e Instituto de Estudios de la Inmunidad Humoral (IDEHU), CONICET, Universidad de Buenos Aires, Buenos Aires, Argentina; f Departamento de Ciencias Biológicas, Facultad de Farmacia y Bioquímica, Universidad de Buenos Aires, Buenos Aires, Argentina; g Gastroenterology Department, Hospital Italiano de Buenos Aires, Buenos Aires, Argentina; h Departamento de Patología, Hospital de Clínicas “José de San Martín”, Universidad de Buenos Aires, Argentina; i Bernardino Rivadavia Hospital, Buenos Aires, Argentina; j Unidad de Investigaciones Biomédicas en Cáncer (IBioCAN), Centro de Medicina Traslacional (CEMET), Hospital El Cruce (HEC), Buenos Aires, Argentina; k Centro de Oncología Molecular y Traslacional (COMTra), Universidad Nacional de Quilmes (UNQ), Buenos Aires, Argentina; l Instituto de Investigaciones Farmacológicas (ININFA), Facultad de Farmacia y Bioquímica, Universidad de Buenos Aires - CONICET, Buenos Aires, Argentina; m Cátedra de Inmunología, Departamento de Microbiología, Inmunologia, Biotecnología y Genética, Facultad de Farmacia y Bioquímica, Universidad de Buenos Aires, Argentina; n Centro de Investigaciones en Biomedicina Traslacional (CIBiMeT), Hospital de Alta Complejidad del Bicentenario Esteban Echeverría - CONICET, Argentina; o Instituto de Investigaciones en Ciencias de la Salud, Consejo Nacional de Investigaciones Científicas y Técnicas and Facultad de Ciencias Médicas-Universidad Nacional de Córdoba, Ciudad Universitaria, 5000, Córdoba, Argentina; p Departamento de Cirugia, Hospital de Clínicas “José de San Martín”, Universidad de Buenos Aires, Argentina; q Department of Surgical Oncology, Institut Paoli-Calmettes, Marseille, France; r Aix-Marseille Univ, INSERM U1068, CNRS UMR 7258, Institut Paoli-Calmettes, Centre de Recherche en Cancérologie de Marseille (CRCM), Parc Scientifique et Technologique de Luminy, Marseille, France; s Department of Clinical Oncology, Institut Paoli-Calmettes, Marseille, France; t General Surgery Department, Hospital Italiano de Buenos Aires, Buenos Aires, Argentina

**Keywords:** Pancreatic ductal adenocarcinoma (PDAC), Stage-dependent therapeutic vulnerability, Pathway rewiring, Metabolic reprogramming, Precision oncology

## Abstract

Pancreatic ductal adenocarcinoma (PDAC) remains clinically managed largely through anatomical stage and performance status, despite major inter-patient heterogeneity and dynamic pathway adaptation during progression. In this review, we propose a hypothesis-generating, stage-aware framework in which clinical stage and established molecular subtypes are considered complementary rather than competing stratification axes. Stage-resolved transcriptomic modeling identified a directional separation across up-front resectable, borderline/locally advanced, primary metastatic and liver metastatic samples, with PC1 explaining 51.1% of the variance and separating early from metastatic states, whereas PC2 explained 30.2% and captured immune/stromal remodeling. We interpret these observations cautiously as pathway-level signals that require functional and clinical validation, not as an established biological paradigm. The model suggests a transition from MAPK-enriched, proliferative and partially immune-competent tumors toward metastatic ecosystems characterized by PI3K-AKT-mTOR engagement, MYC amplification, mitochondrial metabolism, proteostasis support and immune exclusion. We further distinguish approved standards, negative clinical experiences, early clinical signals and preclinical hypotheses. The central conclusion is that PDAC therapeutic stratification should integrate stage, molecular subtype, treatment line, tissue context and evidence level before pathway-directed interventions are clinically prioritized. Accordingly, this review should be read as a translational prioritization framework rather than as a treatment algorithm: it organizes where pathway-directed hypotheses are most biologically plausible, where they are clinically supported, and where they remain insufficiently validated for routine decision-making.

## Highlights

• PDAC progression reflects stage-specific pathway rewiring rather than uniform disease escalation.
• Resectable PDAC is defined by MAPK-driven proliferation, glycolytic metabolism, and partial immune competence.
• Borderline/locally advanced PDAC represents a transitional state with emerging PI3K-AKT-mTOR activation and metabolic plasticity.
• Metastatic PDAC converges toward mitochondrial metabolism, redox adaptation, proteostasis support, and immune evasion.
• Stage-aware therapeutic stratification may improve precision treatment by aligning drugs with dominant pathway dependencies.

## Graphical abstract

**Figure:**
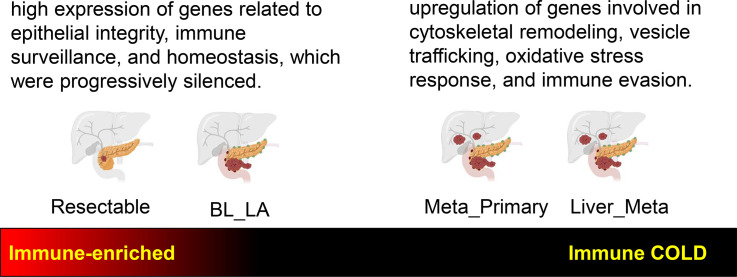

## Introduction

Pancreatic ductal adenocarcinoma (PDAC) remains one of the most lethal malignancies worldwide, characterized by early dissemination, extensive stromal remodeling, and profound resistance to therapy. Despite advances in genomic characterization, PDAC continues to be treated largely as a single disease entity, with therapeutic strategies primarily guided by anatomical stage rather than molecular dependencies. This approach overlooks the dynamic biological evolution of PDAC and the progressive activation of distinct signaling, metabolic, and immune-regulatory pathways that may define stage-specific vulnerabilities [1,2]. A key gap addressed in this review is not the absence of molecular classifications in PDAC, but their incomplete integration with clinical stage, metastatic site, treatment line and pathway-level therapeutic decision-making. Collisson [3], Moffitt [4], Bailey [5] and PurIST [6] classifications have established that PDAC contains reproducible transcriptional states, including classical/progenitor-like and basal-like/squamous programs, as well as tumor- and stroma-associated subtypes. However, these systems were primarily designed to capture intrinsic tumor biology, stromal composition or single-sample subtype assignment, rather than to define how dominant pathway outputs may shift across clinically relevant stages. Consequently, molecular subtype and anatomical stage should not be regarded as interchangeable variables. A resectable tumor may display basal-like features, and a metastatic tumor may retain classical characteristics; conversely, treatment exposure, tumor purity and sampling site can modify apparent subtype assignment. The framework proposed here therefore aims to add a second, clinically anchored axis to existing subtype models, rather than to replace them. The conceptual contribution of this review is therefore integrative rather than taxonomic. We do not propose another subtype classifier; instead, we use clinical stage and metastatic context as an organizing scaffold to interpret how established tumor-intrinsic subtypes, stromal states, immune landscapes, pathway outputs and therapeutic evidence may intersect in practice. This distinction is essential because a clinically useful model must help prioritize trials and biomarkers, not merely describe biological diversity.

Cancer development is traditionally divided into transformation and progression. Transformation involves oncogenic mutations, most notably KRAS activation, along with alterations in tumor suppressors and epigenetic regulators that initiate malignant growth. However, progression represents a distinct biological phase, characterized by coordinated transcriptional reprogramming, metabolic rewiring, immune remodeling, and microenvironmental adaptation [7]. These processes evolve across clinically defined stages rather than remaining static. Early-stage PDAC frequently retains partial epithelial identity, residual immune activation, and reliance on proliferative and glycolytic programs. In contrast, advanced and metastatic lesions often exhibit enhanced PI3K-AKT-mTOR signaling, MYC-driven transcriptional amplification, cytoskeletal remodeling, oxidative phosphorylation dependence, and an immunocompromised microenvironment [8]. These shifts suggest that PDAC progression involves sequential engagement of distinct oncogenic circuits. Current treatment strategies rarely integrate pathway-level heterogeneity into clinical decision-making. Cytotoxic chemotherapy remains the backbone of therapy across all stages, while targeted and immunotherapeutic approaches have demonstrated limited efficacy, potentially because patients are not stratified according to activated molecular programs [9,10]. If PDAC progression involves dynamic shifts in dominant signaling and metabolic dependencies, then therapeutic strategies should reflect this biology. Early-stage tumors characterized by replication stress and nucleotide biosynthetic activity may preferentially benefit from DNA-damaging agents and antimetabolites. Conversely, advanced tumors exhibiting mitochondrial metabolism, redox adaptation, and PI3K-AKT-mTOR activation may require metabolic inhibitors or pathway-targeted combinations. Likewise, immunotherapeutic approaches should be tailored according to antigen presentation capacity and the underlying immunological landscape [8].

A critical unmet need in PDAC management is the development of stage-aware, pathway-informed therapeutic stratification. Rather than viewing progression solely as clinical worsening, it should be understood as a transition between distinct molecular ecosystems, each potentially harboring actionable vulnerabilities. Dissecting the transcriptional and signaling programs that define these transitions may enable rational therapeutic diversification. In this review, we discuss emerging evidence supporting pathway-driven stratification in PDAC, emphasizing how stage-dependent activation of oncogenic, metabolic, and immune programs can inform therapeutic decision-making. By integrating transcriptomic, functional, and clinical data, we propose a conceptual framework in which PDAC is treated not as a uniform entity, but as a dynamic disease requiring biologically tailored intervention strategies.

### Analytical framework and quantitative definition of stage-specific rewiring

To systematically characterize stage-dependent biological specialization, we implemented an ensemble-based transcriptomic modeling strategy integrating all available clusters across clinical stages. Expression values were normalized and stage-specificity scores were computed as the differential expression of each gene relative to the mean expression across the remaining stages. This approach enables identification of hierarchical pathway dominance rather than absolute expression differences alone. Importantly, this analytical strategy is intended to generate a structured biological hypothesis. Bulk transcriptomic data can indicate coordinated pathway activity, but it does not directly measure kinase activity, protein abundance, post-translational modifications, tumor-cell-intrinsic versus stromal contributions, or treatment-induced adaptation. For this reason, stage-specificity scores were used as a ranking and interpretation tool, not as proof of causal dependency. A pathway enriched in one stage should be considered a candidate vulnerability only when it can be connected to independent evidence, such as phospho-protein activation, functional perturbation, pharmacodynamic modulation, matched tissue validation or clinical enrichment by a reproducible biomarker.

**Fig. 1:**
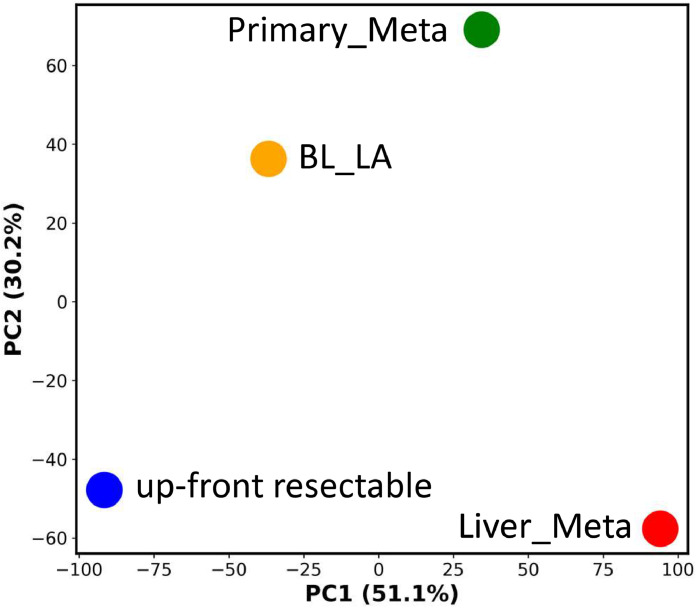
**Stage-resolved principal component analysis reveals structured transcriptomic divergence across PDAC progression.** Principal component analysis (PCA) was performed using differential gene expression values across all clusters and stages. Each point represents the aggregated transcriptomic profile of a clinical stage: up-front resectable, BL/LA, Primary_Meta, and Liver_Meta. PC1 captures the major axis of variation and reflects progressive separation from early, proliferation-dominant tumors toward metabolically specialized metastatic states. PC2 represents an orthogonal dimension associated with immune and stromal remodeling. The spatial distribution of stages supports a directional model of biological refinement across progression rather than stochastic transcriptional drift. The RNA-sequencing data used in this analysis were obtained from publicly available previously published cohort at (https://doi.org/10.5281/zenodo.18619250).

Global divergence between stages was quantified using Euclidean distance matrices and principal component analysis (PCA). Euclidean modeling demonstrated progressive transcriptomic separation along the trajectory up-front resectable → BL/LA → Primary_Meta → Liver_Meta, with liver metastases representing the most transcriptionally distant state from resectable tumors (Fig. 1). Importantly, BL/LA tumors consistently occupied an intermediate position in multidimensional space, supporting their biological role as a transitional state rather than a mere anatomical extension of early disease. PCA further resolved the structure of this divergence. Principal component 1 (51.1% of variance) defined a directional axis, separating up-front resectable tumors from metastatic lesions, consistent with a shift from MAPK-driven proliferative programs toward survival-oriented, mitochondrially specialized states enriched for PI3K-AKT-mTOR signaling and oxidative metabolism. This axis suggests progressive refinement of metabolic and signaling dependencies rather than simple quantitative escalation. Principal component 2 (30.2% of variance) captured an orthogonal dimension characterized by immune and stromal remodeling. BL/LA and Primary_Meta tumors displayed elevated positioning along this axis, consistent with transitional immune reshaping and microenvironmental adaptation, whereas liver metastases diverged, reflecting convergence toward a metabolically specialized and immune-evasive phenotype. Notably, liver metastases exhibited reduced overall transcriptomic dispersion yet amplified expression of stage-specific programs, indicating selective pathway intensification rather than generalized activation. Collectively, these quantitative analyses support a model in which PDAC progression reflects structured, hierarchical pathway refinement rather than stochastic transcriptional drift.

The proposed trajectory should also not be interpreted as a strictly linear evolutionary sequence followed by every patient. Rather, it summarizes a population-level ordering of pathway states across clinically sampled categories. Inter-patient heterogeneity, tumor purity, stromal abundance, metastatic-site selection, prior treatment exposure and biopsy timing can shift an individual tumor away from the average position of its anatomical stage. This limitation is central to the interpretation and supports the need for integrated stage-by-subtype stratification rather than stage-only therapeutic assignment. Operationally, the model should therefore be applied as a stage-by-subtype matrix. For example, a basal-like resectable tumor, a classical metastatic primary tumor and a liver metastasis with strong stromal contamination may occupy different therapeutic and biological positions even if one pathway signature appears dominant in bulk RNA. This is why the revised framework repeatedly separates population-level trends from patient-level decision rules.

### Relationship between clinical stage and established PDAC molecular subtypes

Established PDAC classifications provide an essential context for the present framework. Collisson and colleagues[3] described classical, quasi-mesenchymal and exocrine-like transcriptional programs; Moffitt and colleagues[4] separated tumor-intrinsic classical/basal-like states from normal/activated stromal compartments; Bailey and colleagues[5] proposed squamous, pancreatic progenitor, immunogenic and ADEX subtypes; and PurIST[6] later provided a clinically robust single-sample classifier for basal-like versus classical tumor identity. These systems converge on the concept that PDAC transcriptional identity is not uniform, but they differ in how they account for tumor purity, stromal signal, immune infiltration and platform-specific effects. The stage-aware model proposed here is therefore best understood as orthogonal to subtype assignment. It asks whether, within or across these established subtype backgrounds, dominant pathway outputs and therapeutic vulnerabilities are enriched at different clinical stages or metastatic contexts. This distinction is clinically important. Basal-like/squamous programs are frequently associated with poor prognosis and treatment resistance, but they are not synonymous with metastatic stage. Similarly, classical tumors can progress and metastasize, and stromal-rich samples may appear biologically advanced because of microenvironmental composition rather than tumor-cell-autonomous rewiring. A practical stratification model should therefore combine: i/ anatomical stage and metastatic site, ii/ tumor-intrinsic subtype, iii/ stromal/immune state, iv/ treatment history, and v/ direct pathway activity or surrogate biomarkers.

### Pathways characterizing up-front resectable PDAC and their distinction from advanced stages

Up-front resectable PDAC represents a biologically distinct stage within the continuum of disease progression. Transcriptomic analyses across resectable, borderline/locally advanced, primary metastatic, and liver metastatic tumors reveal that early-stage PDAC is characterized by specific pathway activation states that differentiate it from more advanced disease. The following sections summarize the key pathways that most strongly define up-front resectable PDAC and contrast them with those predominant in later stages [[3], [4], [5]].

Up-front resectable PDAC tumors are enriched in transcriptional programs associated with epithelial identity, cell-cell adhesion, and cytoskeletal organization. A specific gene expression signature characterizes resectable tumors [8]. These include pathways related to cadherin-mediated adhesion, spectrin-based cytoskeletal architecture, and maintenance of epithelial homeostasis. The preservation of these programs suggests limited epithelial-to-mesenchymal transition (EMT) activation in early-stage tumors, distinguishing them from metastatic lesions where these pathways are suppressed. Resectable PDAC exhibits enrichment of immunologically active programs, including cytokine signaling and antigen presentation machinery. Elevated expression of IL15, IL21, and IL17A, together with preserved B2M levels, suggests maintenance of MHC-I stability and features consistent with adaptive immune engagement. Immune deconvolution analyses reveal increased abundance of CD8+ T cells, dendritic cells, and M1 macrophages in early-stage tumors. In contrast, advanced and metastatic stages exhibit features of immune dysfunction, including reduced antigen presentation signatures and enrichment of regulatory or immunomodulatory signals such as CCR4. In up-front resectable PDAC, KRAS downstream signaling is primarily transmitted through the RAF-MEK-ERK (MAPK) cascade. Genes such as RAF1, BRAF, and MAPK1 show early activation, establishing a proliferative baseline. However, there is limited enrichment of the PI3K-AKT-mTOR axis compared with metastatic disease. This relative dominance of MAPK-driven mitogenic signaling, without strong metabolic reprogramming through PI3K-AKT-mTOR, distinguishes up-front resectable tumors from advanced stages where survival and metabolic adaptation pathways become predominant. Metabolic flux inference indicates that early-stage clusters are enriched in glycolysis, pentose phosphate pathway activity, and nucleotide biosynthesis. These pathways support proliferative growth and replication stress, aligning with increased expression of MYC targets involved in DNA replication (MCM family, PCNA, RRM1/2). In contrast, late-stage PDAC shifts toward oxidative phosphorylation, fatty acid β-oxidation, and amino acid degradation, reflecting enhanced mitochondrial dependence in metastatic disease. up-front resectable tumors demonstrate comparatively lower activation of mitochondrial electron transport chain components and oxidative stress response genes (e.g., COQ7, COQ9, EPAS1). This contrasts with advanced PDAC, where oxidative phosphorylation and redox balance pathways are strongly upregulated. The absence of pronounced mitochondrial adaptation in resectable tumors further differentiates them from metastatic lesions.

Collectively, up-front resectable PDAC is characterized by preserved epithelial differentiation, active immune surveillance, predominant MAPK-driven proliferation, and glycolytic/anabolic metabolism, with limited activation of mitochondrial oxidative and PI3K-AKT-mTOR survival pathways. These features distinguish up-front resectable tumors from advanced and metastatic disease, which exhibit immune dysfunction, cytoskeletal remodeling, metabolic flexibility, and enhanced oxidative phosphorylation. Recognizing these pathway-level distinctions provides a biological framework for stage-specific therapeutic stratification.

### Pathways characterizing borderline and locally advanced PDAC

Borderline and locally advanced (BL/LA) PDAC represent an intermediate biological state between resectable and metastatic disease. Transcriptomic profiling across disease stages indicates that BL/LA tumors are not simply anatomically advanced but display distinct pathway activation patterns that reflect transitional signaling, metabolic adaptation, and progressive immune remodeling. The following sections summarize the pathways that most clearly define BL/LA PDAC and distinguish this stage from both operable and metastatic tumors [[11], [12], [13]].

Compared with resectable PDAC, BL/LA tumors exhibit a measurable reduction in genes related to epithelial integrity, cadherin-mediated adhesion, and cytoskeletal stability [8]. However, these programs are not fully suppressed as observed in metastatic disease. This intermediate pattern suggests partial activation of EMT and structural remodeling pathways, reflecting increased invasiveness while retaining elements of epithelial identity. BL/LA PDAC demonstrates early activation of the PI3K-AKT-mTOR axis compared with resectable tumors. While MAPK signaling remains active, there is increasing expression of components associated with survival signaling, biosynthetic growth, and nutrient sensing. This shift represents a transition from predominantly proliferative MAPK-driven signaling toward combined proliferative and survival/metabolic signaling, foreshadowing the strong PI3K-AKT-mTOR enrichment observed in metastatic stages. MYC target genes involved in DNA replication, ribosome biogenesis, and cell-cycle regulation show progressive upregulation in BL/LA tumors. Increased expression of replication licensing factors (MCM family), PCNA, and nucleotide biosynthetic genes indicates heightened replication stress and biosynthetic demand. This proliferative amplification distinguishes BL/LA PDAC from resectable tumors, while remaining less metabolically reprogrammed than fully metastatic disease. Metabolic pathway inference suggests that BL/LA tumors occupy a transitional metabolic state. Glycolytic and pentose phosphate pathway activity remain prominent; however, there is emerging activation of oxidative phosphorylation (OXPHOS) and redox-related pathways compared with resectable PDAC. This hybrid metabolic phenotype indicates increasing metabolic flexibility without the full mitochondrial dependence characteristic of metastatic lesions. Immune deconvolution analyses reveal that BL/LA PDAC begins to lose the immune-activation signatures observed in up-front resectable tumors. This is reflected by a reduction in CD8⁺ T-cell and dendritic cell signatures, accompanied by increasing stromal and fibroblast-associated signals. Although antigen presentation pathways are not completely suppressed, there is evidence of early immune evasion, positioning BL/LA tumors in an intermediate immunological state between resectable PDAC and metastatic disease characterized by profound immune dysfunction. BL/LA tumors demonstrate increasing expression of genes involved in cytoskeletal reorganization and cell motility, including early activation of Rho-GTPase-related pathways. While these programs are fully amplified in metastatic disease, their incremental activation in BL/LA PDAC reflects enhanced local invasion and tissue remodeling, consistent with vascular and perineural involvement typical of this stage.

Borderline and locally advanced PDAC are characterized by partial loss of epithelial programs, progressive activation of PI3K-AKT-mTOR signaling, amplification of MYC-driven proliferative pathways, emerging mitochondrial metabolic engagement, early immune suppression, and increased cytoskeletal remodeling. These features define BL/LA tumors as a biologically transitional state, distinct from immune-active, glycolysis-dominant resectable tumors and from fully metabolically adapted, immune-suppressed metastatic disease. Understanding this intermediate signaling configuration provides a framework for therapeutic strategies specifically tailored to BL/LA PDAC.

### Pathways characterizing primary tumors from metastatic PDAC patients

Primary tumors obtained from patients diagnosed with metastatic PDAC (Primary_Meta) represent clinically advanced disease and display pathway activation patterns consistent with metastatic competence. In the stage-resolved transcriptomic framework, Primary_Meta samples show coordinated upregulation of metastatic-associated transcriptional programs and suppression of early-stage immune and epithelial homeostasis modules [6,14]. Below, we summarize the pathways that most strongly define Primary_Meta tumors and distinguish them from resectable and borderline/locally advanced disease, while highlighting their similarities to liver metastases [8].

Primary_Meta tumors exhibit strong activation of a coherent gene module that increases monotonically across stages. At the stage level, expression of several genes is markedly higher in Primary_Meta than in resectable and BL/LA tumors, and is comparable to liver metastases, consistent with a shared advanced transcriptional state. This program encompasses pathways linked to invasion, survival under stress, and immune evasion, positioning Primary_Meta as a biologically metastatic phenotype even at the primary site. A defining feature of Primary_Meta tumors is enrichment of pathways supporting motility and invasion. Upregulated genes implicated in actin and microtubule dynamics (e.g., RAC3, MAP7D3) suggest activation of Rho-GTPase signaling and cytoskeletal remodeling. This aligns with a pro-invasive phenotype and represents a key distinction from earlier stages, where epithelial maintenance programs are comparatively preserved. Primary_Meta tumors show increased expression of genes involved in vesicle trafficking and organelle homeostasis (e.g., COPZ2, RAB31, BNIP1), consistent with adaptation to heightened secretory and biosynthetic demands. These pathways may facilitate membrane turnover, receptor recycling, and stress tolerance, processes commonly associated with metastatic progression. Pathways linked to hypoxia adaptation and angiogenic signaling are prominent in Primary_Meta, including induction of EPAS1 (HIF-2α) and related oxygen-sensing programs. In parallel, redox homeostasis pathways are reinforced by increased expression of genes involved in antioxidant capacity and mitochondrial cofactor biosynthesis (e.g., SEPHS1, COQ7). These features are consistent with adaptation to hypoxic, nutrient-limited microenvironments and increased oxidative stress. While KRAS signaling is active across PDAC stages, Primary_Meta tumors display increased engagement of the PI3K-AKT-mTOR axis (e.g., PIK3CA/PIK3CB, AKT3, MTOR), a pattern that becomes more evident in metastatic disease compared with resectable and BL/LA tumors. This pathway supports survival signaling, biosynthetic growth, and metabolic adaptation, and is a central hallmark differentiating metastatic-stage primary tumors from earlier stages dominated by MAPK-driven proliferative output. Primary_Meta tumors show strong MYC target engagement, reflecting a transcriptional amplification state that supports rapid proliferation and anabolic capacity. Enriched programs include replication licensing (MCM family), DNA synthesis and repair (PCNA, RRM1/2), cell-cycle progression (CDK4/6, cyclins), and increased ribosome biogenesis/translation. This MYC-driven architecture likely cooperates with KRAS pathway rewiring to sustain high proliferative turnover in advanced disease. Metabolic pathway inference indicates that late-stage tumors, including Primary_Meta, are enriched in OXPHOS, fatty acid β-oxidation, and amino acid degradation pathways, marking a transition from the glycolytic and pentose phosphate dominance typical of earlier stages. This mitochondrial shift is consistent with enhanced metabolic flexibility and may create vulnerabilities to OXPHOS and redox-targeted interventions in the metastatic setting. Primary_Meta tumors also activate stress-survival programs that support proteome integrity and adaptation to metabolic duress, including ubiquitin/proteasome- and ER-associated degradation-related pathways. Upregulation of factors such as USP9X and NUAK1 suggests reinforcement of proteostasis control and energy-stress signaling, which may contribute to therapy resistance. Compared with resectable tumors, Primary_Meta samples exhibit marked attenuation of immune activation signals, including reduced expression of effector cytokines (e.g., IL15, IL17A, IL21) and decreased expression of antigen presentation-related components such as B2M, coupled with increased expression of immunoregulatory mediators including CCR4 and CXCL16. Immune deconvolution analyses across stages indicate a progressive reduction CD8+ T cells, dendritic cells, and M1 macrophages with advancing disease, consistent with the emergence of an immune-excluded and immunoregulatory microenvironment in metastatic-stage tumors. Primary_Meta tumors display activation of extracellular matrix (ECM) remodeling and stromal interaction pathways. Upregulation of LOXL1 and additional stromal/vascular-associated factors (e.g., ANGPTL2, FBN1, CAV1, S1PR3) supports a microenvironment conducive to invasion and dissemination, and aligns with increased desmoplasia and vascular signaling typical of advanced PDAC.

Primary tumors from metastatic PDAC patients are defined by: i/ strong activation of metastatic adaptation programs (invasion/motility, vesicular trafficking, hypoxia/redox responses), ii/ oncogenic signaling rewiring favoring PI3K-AKT-mTOR and sustained MYC target engagement, iii/ metabolic progression toward mitochondrial oxidative metabolism and stress tolerance, and iv/ immune suppression with stromal expansion and ECM remodeling. Together, these pathways distinguish Primary_Meta tumors from resectable and BL/LA disease and support a framework in which metastatic-stage primaries are treated using pathway-informed strategies rather than anatomy-driven escalation alone.

### Pathways characterizing liver metastases in PDAC

Liver metastases (Liver_Meta) represent the terminal and most biologically adapted state within the PDAC progression continuum. Compared with resectable, borderline/locally advanced, and even primary tumors from metastatic patients (Primary_Meta), hepatic metastases display the most pronounced activation of metastatic, metabolic, and immune-evasive programs. These lesions reflect not only advanced tumor-intrinsic evolution but also adaptation to the hepatic microenvironment, which imposes unique metabolic and immunological pressures.

Liver_Meta samples show the highest expression levels of several genes, representing the culmination of the monotonic transcriptional program activated during progression [8]. This module includes pathways linked to invasion, stress tolerance, immune evasion, and metabolic flexibility. Compared with Primary_Meta tumors, liver metastases exhibit further amplification of these pathways, consistent with full metastatic competence. Genes involved in actin cytoskeleton regulation and Rho-GTPase signaling (e.g., RAC3, LIMK1-related pathways) are strongly upregulated in Liver_Meta. This supports sustained motility, colonization capacity, and structural plasticity. The persistent activation of these programs likely facilitates adaptation to hepatic sinusoidal architecture and microvascular invasion. Liver metastases demonstrate strong activation of hypoxia-responsive pathways, including EPAS1 (HIF-2α)-associated signaling. These pathways regulate angiogenesis, metabolic adaptation, and survival under oxygen-limited conditions. The hepatic microenvironment, characterized by fluctuating oxygen gradients and high metabolic turnover, may further select for tumors capable of robust hypoxia adaptation. Among all stages, Liver_Meta exhibits the strongest enrichment of OXPHOS, fatty acid β-oxidation, and amino acid degradation pathways. Upregulation of electron transport chain components (e.g., COQ7, COQ9, COX6A1, NDUFA13) and redox-balancing enzymes reflects heightened mitochondrial dependence. This metabolic configuration supports energy flexibility and survival in nutrient-variable hepatic niches. Liver_Meta tumors show reinforced engagement of the PI3K-AKT-mTOR axis relative to earlier stages. This pathway promotes anabolic growth, nutrient sensing, and resistance to metabolic stress. Compared with resectable and BL/LA tumors, this survival signaling branch becomes dominant in metastatic liver lesions. Enhanced expression of genes involved in ER-associated degradation (ERAD), ubiquitin-proteasome pathways, and deubiquitinases (e.g., USP9X) indicates adaptation to proteotoxic stress. These programs may contribute to resistance against oxidative damage and therapy-induced stress in metastatic settings.

Liver_Meta lesions exhibit the most pronounced immune dysfunction across stages. These tumors show marked downregulation of cytokines associated with cytotoxic and adaptive immune responses (e.g., IL15 and IL21), along with reduced expression of antigen presentation-related components such as B2M. This is accompanied by enrichment of immunoregulatory and chemokine signaling pathways, including CCR4 and CXCL16. Immune deconvolution analyses indicate decreased CD8+ T cells and dendritic cell signatures, together with increased stromal and myeloid-associated components. Given the intrinsically tolerogenic nature of the hepatic microenvironment, these lesions may exploit local immune regulatory circuits to facilitate immune evasion. Extracellular matrix (ECM) remodeling genes (e.g., LOXL1) and vascular/stromal interaction factors (ANGPTL2, FBN1, CAV1, S1PR3) are strongly expressed in Liver_Meta. These pathways likely facilitate colonization, matrix stiffening, angiogenic remodeling, and integration into the hepatic microenvironment.

Liver metastases represent the apex of PDAC progression and are characterized by: i/ maximal activation of metastatic adaptation modules, ii/: dominant mitochondrial oxidative metabolism and redox control, iii/ reinforced PI3K-AKT-mTOR survival signaling, iv/ robust cytoskeletal and invasive programs, and v/ profound immune dysfunction within a tolerogenic hepatic niche. These pathways distinguish Liver_Meta not only from resectable and BL/LA disease but also from Primary_Meta tumors, defining a fully metabolically adapted and immune-evasive metastatic state.

### Stage-dependent pathway rewiring in PDAC: therapeutic implications and drug mapping

The therapeutic mapping proposed below has been to avoid placing approved agents, negative clinical experiences, early-phase signals and preclinical concepts on the same evidentiary level. For each stage, clinically validated chemotherapy backbones remain the reference standard unless a validated biomarker identifies a specific alternative. Pathway-directed strategies are discussed as trial priorities or biological hypotheses, not as established treatment recommendations. This distinction is particularly important in PDAC, where several rational approaches have failed to improve outcomes when tested clinically, including stromal depletion or hyaluronan targeting in broad or biomarker-selected populations, single-agent MEK inhibition strategies, immune checkpoint blockade in unselected microsatellite-stable disease, and some mitochondrial/metabolic approaches such as devimistat combined with modified FOLFIRINOX in phase III testing [15,16]. These negative experiences do not invalidate pathway biology, but they demonstrate that target presence, pathway activation and clinical efficacy are not equivalent. We therefore propose a therapeutic-readiness matrix. Standard-of-care strategies should be separated from: i/ molecularly selected approved or near-approved approaches, such as MSI-H/dMMR-directed immune checkpoint blockade[17], germline BRCA/PALB2-associated platinum sensitivity and PARP maintenance [16], NTRK fusions, and rare KRAS G12C-directed strategies [18]; ii/ early clinical concepts, including selected EGFR-RAS-MAPK or KRAS-allele-specific combinations; and iii/ preclinical or mechanistic concepts, including OXPHOS inhibition, FAO targeting, BET inhibition, CCR4/CSF1R modulation, proteostasis stress amplification and redox disruption. Table 1, Table 2 should explicitly indicate clinical status, evidence strength, biomarker requirements and unresolved controversies for each therapeutic domain.

**Table 1:** Stage-dependent biological architecture in PDAC: evidence-aware framework.

Domain	Up-front resectable	BL/LA	Primary tumor in metastatic PDAC	Liver metastasis	Evidence, caveats and validation needs
**Position relative to subtype systems**	Often enriched for epithelial/classical features, but not equivalent to any subtype.	Mixed states; classical–basal plasticity may coexist with local invasion.	Higher probability of basal-like/squamous traits, but variable across patients.	Metastatic-site selection may enrich stress-adapted programs.	Stage and subtype are complementary, not competing. Collisson/Moffitt/Bailey/PurIST classify tumor states; stage-aware analysis asks how dependencies shift over clinical progression.
**Dominant signaling output**	KRAS output mainly MAPK/RAF–MEK–ERK; proliferative and epithelial maintenance programs.	MAPK persists with emerging PI3K–AKT–mTOR, TGF-β and motility circuits.	PI3K–AKT–mTOR, MYC, hypoxia and survival signaling become more prominent.	Reinforced survival, Rho/cytoskeletal and stress-response signaling.	Transcript levels do not prove pathway activity. Phosphoproteomic/proteomic validation is needed to explain signaling rewiring despite stable drivers.
**Metabolic adaptation**	Glycolysis, PPP, nucleotide synthesis and replication stress.	Hybrid metabolism: glycolysis maintained with emerging OXPHOS/redox programs.	Greater OXPHOS, FAO, amino-acid use and redox dependence.	Mitochondrial specialization, FAO/redox buffering and hepatic niche adaptation.	Bulk transcriptomics cannot define substrate flux. Functional assays, isotope tracing and spatial context remain required.
**Immune and stromal context**	Partial antigen presentation and immune competence may persist.	Early immune erosion; CAF/TGF-β and ECM signals increase.	Immune suppression, myeloid/Treg enrichment and stromal exclusion.	Tolerogenic hepatic niche with immune exclusion and vascular/ECM adaptation.	Deconvolution is affected by tumor purity, sampling and stromal abundance. Single-cell/spatial validation is essential.
**Adaptive resistance mechanisms**	DNA repair tolerance, residual clones and compensatory survival signaling.	EMT plasticity, stromal protection and metabolic switching.	Proteostasis, UPR, epigenetic switching, PTM/phosphorylation rewiring.	Redox buffering, drug-tolerant states, niche protection and immune escape.	Resistance is not only transcriptional. Post-transcriptional, post-translational and epigenetic mechanisms should be explicitly included.
**Biomarker readiness**	Standard markers: BRCA/PALB2, MSI/dMMR, NTRK/rare fusions; subtype assays exploratory.	Tissue adequacy, treatment history and re-biopsy feasibility become limiting.	ctDNA, RNA subtype, phospho-signaling and immune/stromal markers may guide trials.	Metastatic biopsy site and liver-specific microenvironment must be considered.	Clinical implementation requires assay thresholds, turnaround time, tissue source, treatment line and prospective validation.

This table summarizes a stage-resolved interpretation of the biological architecture of PDAC, integrating tumor-cell programs, signaling dependencies, metabolic adaptations, immune/stromal context, resistance mechanisms and biomarker readiness across up-front resectable disease, borderline/locally advanced tumors, primary tumors from metastatic PDAC and liver metastases. The framework emphasizes that clinical stage and molecular subtype are complementary rather than interchangeable dimensions: subtype systems capture tumor-state identity, whereas a stage-aware approach highlights how biological dependencies and therapeutic vulnerabilities may shift during disease progression. The final column outlines key evidentiary caveats and validation needs, including the limitations of transcriptomic inference, the need for proteomic/phosphoproteomic and functional metabolic validation, the influence of tumor purity and stromal abundance, and the importance of prospective biomarker assessment before clinical implementation.

Abbreviations: BL/LA, borderline/locally advanced; PPP, pentose phosphate pathway; FAO, fatty-acid oxidation; UPR, unfolded protein response; PTM, post-translational modification; CAF, cancer-associated fibroblast; ECM, extracellular matrix; ctDNA, circulating tumor DNA; MSI/dMMR, microsatellite instability/deficient mismatch repair.

**Table 2:** Stage-aware therapeutic stratification in PDAC: evidence level and clinical caveats.

Clinical context	Validated backbone / current role	Biomarker-defined options	Trial-level / experimental rationale	Negative evidence or caution	Practical implementation
**Up-front resectable**	Surgery when feasible; adjuvant mFOLFIRINOX in fit patients; gemcitabine-based alternatives when needed.	BRCA/PALB2 may support platinum sensitivity; MSI/dMMR, NTRK/rare fusions are uncommon but actionable.	Neoadjuvant intensification, vaccines/CD40 or immune priming should remain trial-based.	Stage alone should not justify targeted therapy; MEK/PI3K or immunotherapy in unselected PDAC is not established.	Obtain high-quality tissue before therapy when possible; record subtype, germline/somatic testing and surgical timing.
**Borderline / locally advanced**	FOLFIRINOX or gemcitabine/nab-paclitaxel-based induction; consolidation chemoradiation in selected cases.	DNA repair defects, MSI/dMMR and rare fusions should be screened; subtype assays remain investigational.	Combination strategies targeting stromal normalization, DNA damage response, MAPK/PI3K or immune priming should be tested prospectively.	Broad stromal depletion and PEGPH20 did not improve survival in phase III; targeted signaling combinations may be toxic or ineffective without biomarkers.	Use multidisciplinary review; reassess resectability; consider repeat biopsy/ctDNA after induction to capture adaptation.
**Primary tumor in metastatic PDAC**	First-line mFOLFIRINOX, gemcitabine/nab-paclitaxel or NALIRIFOX depending on fitness, availability and guidelines.	BRCA/PALB2: platinum/PARP maintenance context; KRAS G12C inhibitors or RAS-directed agents only in genotype-defined settings/trials; MSI/dMMR: checkpoint blockade.	RAS(ON), KRAS G12D, ERK/MEK, PI3K/mTOR, CDK4/6, BET, myeloid/Treg and metabolic combinations are rational but mostly investigational.	Checkpoint inhibitors show little benefit in unselected PDAC; single-agent MEK and many metabolic approaches have limited activity or toxicity.	Treatment line matters: first-line cytotoxic backbone vs later-line genotype-driven trials; integrate ctDNA, biopsy site and performance status.
**Liver metastasis**	Systemic therapy as for metastatic PDAC; later-line choices guided by prior exposure and performance status.	Genotype-driven RAS targeting is emerging; DNA repair/MSI/fusion-directed options remain rare but important.	Mitochondrial, redox, FAO, proteostasis and immune-myeloid/stromal reprogramming strategies are hypothesis-driven and should be combination-trial concepts.	OXPHOS inhibitors have faced dose-limiting toxicity; proteasome/redox strategies lack validated PDAC biomarkers; hepatic immune tolerance may blunt immunotherapy.	Biopsy of the metastatic site may differ from primary tumor; include liver niche, ctDNA dynamics and toxicity monitoring in trial design.

This table summarizes therapeutic opportunities in PDAC according to clinical context, distinguishing validated treatment backbones, biomarker-defined options, trial-level hypotheses, negative clinical evidence and practical implementation constraints. The framework emphasizes that therapeutic assignment should not be based on disease stage alone, but should integrate resectability status, treatment line, patient fitness, molecular genotype, DNA repair status, MSI/dMMR or rare fusion events, tissue availability, biopsy site and prospective biomarker validation. Standard cytotoxic approaches remain the reference backbone across stages, whereas targeted, immune, stromal, metabolic, redox, proteostasis and liver-niche-directed strategies should be interpreted according to their level of evidence and generally prioritized within molecularly selected or well-designed clinical trials. The table also highlights key cautionary areas, including the limited efficacy of immunotherapy in unselected PDAC, the failure of broad stromal depletion strategies, toxicity concerns with some signaling or metabolic combinations, and the need to distinguish validated standards from early-phase or preclinical hypotheses.

Abbreviations: PDAC, pancreatic ductal adenocarcinoma; mFOLFIRINOX, modified folinic acid, fluorouracil, irinotecan and oxaliplatin; NALIRIFOX, liposomal irinotecan, fluorouracil/leucovorin and oxaliplatin; MSI/dMMR, microsatellite instability/deficient mismatch repair; ctDNA, circulating tumor DNA; FAO, fatty-acid oxidation; OXPHOS, oxidative phosphorylation; PARP, poly(ADP-ribose) polymerase.

PDAC progression is accompanied by coordinated rewiring of dominant signaling, metabolic, immune, and stress-adaptation pathways. These biological transitions suggest that therapeutic strategies should be aligned with stage-specific pathway dependencies rather than applied uniformly across disease states. Below, we integrate the conceptual pathway shift along progression model with representative drug classes and therapeutic strategies that may be rationally prioritized at each stage.

### Operable PDAC: proliferation-driven and immune-competent state

Up-front resectable tumors are characterized by MAPK-dominant proliferative signaling, glycolysis and nucleotide biosynthesis enrichment, preserved epithelial identity, and partial immune activation. These features support prioritization of DNA-damaging and antimetabolite-based chemotherapy, including gemcitabine, fluoropyrimidines (5-FU), and platinum agents, which exploit replication stress and nucleotide demand [19,20]. In selected contexts, MEK inhibitors (e.g., trametinib) may target MAPK dependency [21,22]. These findings suggest that early-stage tumors may retain sufficient antigen presentation capacity and cytotoxic immune engagement to support immune-priming interventions, whereas advanced disease is marked by immune exclusion and functional impairment that may limit responsiveness. In the revised interpretation, MEK inhibition and immune-priming strategies in operable PDAC should be considered investigational unless supported by a biomarker-driven trial. The clinical priority remains optimal local control, systemic chemotherapy appropriate to patient fitness, and prospective collection of tissue for subtype and pathway assessment. The apparent MAPK dominance of early tumors provides a biological rationale for studying MAPK-directed combinations, but prior experience in PDAC argues against extrapolating from pathway activation alone to clinical benefit. Resistance in this setting is likely to emerge from residual tumor clones that survive cytotoxic pressure through DNA damage repair proficiency, compensatory PI3K-AKT-mTOR signaling, EMT-like plasticity, autophagy and stromal protection. Therefore, trials in operable PDAC should ideally collect pretreatment biopsies and post-treatment resection specimens to distinguish baseline stage-associated biology from therapy-induced adaptation.

### Borderline/locally advanced PDAC: transitional signaling and early survival engagement

BL/LA tumors exhibit partial epithelial erosion, emerging PI3K-AKT-mTOR activation, hybrid metabolism, and early immune suppression. Combination strategies integrating cytotoxic backbones (e.g., FOLFIRINOX) with pathway-targeted agents may be rational. PI3K inhibitors (e.g., alpelisib) [23], AKT inhibitors (e.g., capivasertib) [24], or mTOR inhibitors (e.g., everolimus) could be explored in biomarker-selected contexts. Metabolic co-targeting strategies combining glycolysis inhibitors with emerging OXPHOS inhibitors (e.g., IACS-010,759) [25] may address transitional metabolic flexibility. Stromal-modulating agents (e.g., PEGPH20 targeting hyaluronic acid in selected tumors) may help counteract increasing stromal exclusion [26]. For BL/LA disease, stage-aware therapeutic stratification must be embedded within neoadjuvant or conversion-treatment decisions. FOLFIRINOX-based or gemcitabine/nab-paclitaxel-based regimens remain the clinical backbone. PI3K-AKT-mTOR, stromal or metabolic co-targeting should be proposed only in molecularly selected trials, because pathway activation is common but predictive biomarkers are not yet validated. Particular caution is required for stromal modulation: the stroma may restrict drug delivery and promote immune exclusion, but it can also restrain tumor dissemination, and indiscriminate stromal depletion has produced disappointing or adverse results in PDAC models and clinical development. For this reason, BL/LA disease is a particularly appropriate setting for adaptive neoadjuvant designs, where serial imaging, CA19-9 kinetics, tissue or liquid-biopsy molecular profiling and surgical conversion endpoints can be linked to pharmacodynamic pathway suppression. The therapeutic question is not simply whether a pathway is active, but whether inhibiting it improves conversion, margin status, recurrence risk or survival beyond modern chemotherapy backbones.

### Primary tumors from metastatic patients: survival and metabolic dominance

Primary_Meta tumors demonstrate strong PI3K-AKT-mTOR engagement, MYC-driven transcriptional amplification, mitochondrial oxidative metabolism enrichment, and immune dysfunction. Therapeutic strategies may prioritize dual pathway blockade (e.g., MEK + PI3K/mTOR combinations), cell-cycle targeting (e.g., CDK4/6 inhibitors such as Palbociclib [27]), and inhibitors of ribosome biogenesis or transcriptional addiction (e.g., BET inhibitors [28]). Metabolic targeting becomes increasingly relevant, including OXPHOS inhibitors (IACS-010,759 [29]), fatty acid oxidation inhibitors (etomoxir [30], experimental), and redox-disrupting agents. Immune reactivation strategies may require combination approaches targeting Treg-mediated and myeloid-driven immunoregulatory mechanisms (e.g., CCR4 antagonists [31], CSF1R inhibitors [32]) alongside checkpoint blockade. Immune reactivation strategies may require combination approaches that include CCR4 antagonists²⁹ or CSF1R inhibitors³⁰ alongside checkpoint blockade.

### Liver metastases: mitochondrial and immune-evasive ecosystem

Liver metastases represent maximal metabolic adaptation and immune exclusion. These tumors show dominant OXPHOS, fatty acid oxidation, reinforced PI3K-AKT-mTOR signaling, and strong redox buffering [8]. Therapeutic strategies may emphasize mitochondrial metabolism inhibitors [29], redox-modulating approaches (e.g., glutathione pathway targeting, pro-oxidant therapies), and combined PI3K-AKT-mTOR blockade. Proteostasis inhibitors (e.g., proteasome inhibitors such as bortezomib or marizomib [33], though historically limited in PDAC) may be reconsidered in combinatorial stress-amplification strategies. Given the tolerogenic hepatic environment, immune strategies may require multi-layered approaches integrating checkpoint blockade with myeloid reprogramming or stromal/vascular targeting [34]. For liver metastases, metabolic specialization and immune exclusion are treated as mechanistic hypotheses with potential therapeutic value, not as immediately actionable biomarkers. The hepatic niche imposes unique metabolic and immunological constraints, but liver biopsy samples may differ from matched primary tumors because of selection, clonal bottlenecks, prior therapy and microenvironmental contamination. Mitochondrial, redox, proteostasis and myeloid/stromal targeting should therefore be prioritized in adaptive clinical trials that include pharmacodynamic endpoints and paired tissue or liquid-biopsy monitoring. Whenever feasible, paired analysis of the primary tumor and metastatic lesion would be preferable, because liver metastases may amplify mitochondrial, lipid-metabolic and immune-tolerant programs that are not dominant in the matched primary site. This paired approach would also help distinguish true metastatic adaptation from sampling effects, stromal dilution and treatment-driven selection.

### Stromal, immune and metabolic targeting in PDAC

Stromal targeting is biologically attractive because desmoplasia can impair drug delivery, promote hypoxia and foster immune exclusion; however, PDAC stroma can also restrain tumor growth or limit dissemination in some contexts. Therefore, the goal should not be indiscriminate stromal depletion, but biomarker-guided stromal remodeling with clear pharmacodynamic and safety endpoints. A similar caution applies to immunotherapy. The presence of immune-related transcripts in early-stage or transitional tumors does not necessarily indicate checkpoint sensitivity, especially in microsatellite-stable PDAC. Effective immune strategies will probably require patient selection, restoration of antigen presentation, modulation of myeloid and fibroblast barriers, and rational combination with chemotherapy, radiation or targeted agents. Metabolic targeting should also be interpreted critically. OXPHOS, fatty-acid oxidation, redox buffering or proteostasis signatures may identify adaptive states, but metabolic plasticity can rapidly bypass single-node inhibition. Metabolic trials should therefore include pharmacodynamic proof of pathway engagement, compensatory pathway monitoring and rational combinations rather than relying solely on transcriptomic enrichment.

## Conclusion

**Fig. 2:**
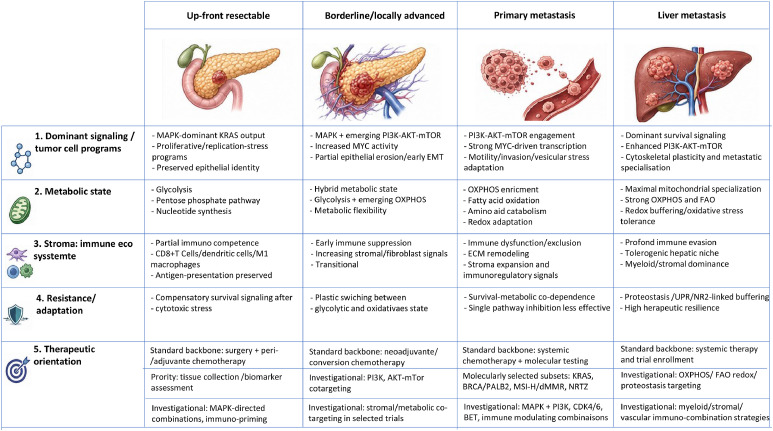
**Stage-resolved biological evolution and therapeutic vulnerabilities in PDAC.** Integrated overview of the proposed progression of PDAC from up-front resectable disease to borderline/locally advanced tumors, primary metastatic disease, and liver metastasis. Across these clinical stages, tumor biology evolves from a predominantly KRAS/MAPK-driven, proliferative and partially epithelial state toward increasingly plastic, invasive and therapeutically resilient programs characterized by PI3K–AKT–mTOR engagement, MYC activity, epithelial erosion, metabolic flexibility, mitochondrial specialization, redox adaptation and proteostasis buffering. This progression is accompanied by a shift from partial immune competence, with preserved antigen presentation and cytotoxic immune components, toward immune dysfunction, stromal expansion, myeloid/stromal dominance and, in liver metastasis, adaptation to a tolerogenic hepatic niche. Metabolically, early glycolytic and biosynthetic programs progressively give way to hybrid and oxidative states enriched in OXPHOS, fatty acid oxidation, amino acid catabolism and oxidative stress tolerance. The lower panel summarizes corresponding therapeutic orientations, emphasizing standard clinical backbones at each stage together with investigational opportunities, including MAPK-directed combinations, PI3K-AKT-mTOR co-targeting, stromal and immune-modulating strategies, metabolic targeting, redox/proteostasis interference and molecularly selected approaches.

This integrative framework illustrates that PDAC progression entails a directional shift from a proliferation-centered, glycolysis-dominant and partially immune-active state toward a metabolically flexible, immune-dysfunction, mitochondrially adapted metastatic ecosystem. Mapping representative drug classes onto stage-specific pathway dependencies highlights the need for biologically stratified therapeutic strategies. Future clinical trial designs should incorporate pathway activation signatures to align therapeutic selection with dominant tumor biology, moving beyond anatomy-based treatment escalation toward precision stage-aware intervention (Fig. 2).

### Biomarker implementation and practical clinical translation

A clinically usable stage-aware framework would require practical biomarker rules rather than descriptive pathway labels. At minimum, patient assignment should integrate anatomical stage, treatment line, biopsy site, tumor purity, molecular subtype, key genomic alterations, and feasible assays for pathway activation. Potential assays include targeted DNA/RNA panels, single-sample subtype classifiers such as PurIST, immunohistochemistry or multiplex immunofluorescence for antigen presentation and immune exclusion, phospho-protein readouts for MAPK/PI3K signaling, and metabolically informative markers when tissue quality permits. However, thresholds for pathway positivity remain undefined for most proposed vulnerabilities, and bulk RNA signatures can be confounded by stromal content, necrosis, inflammation and prior chemotherapy.

Implementation must also be adapted to treatment timing. In resectable or BL/LA disease, pretreatment biopsies may be small and dominated by stroma, whereas resection specimens are often obtained after systemic therapy and may no longer represent the untreated state. In metastatic disease, liver or peritoneal biopsies may better capture the actionable ecosystem but can differ from the primary lesion. Circulating tumor DNA can support genomic selection but does not yet replace tissue-based pathway or microenvironmental assessment. For these reasons, the revised framework is presented as a rationale for biomarker-enriched trial design, serial sampling and adaptive therapeutic algorithms rather than as a ready-to-use clinical decision tree. A pragmatic implementation workflow would include: first, assignment of anatomical stage, metastatic site and treatment line; second, genomic testing for immediately actionable subsets, including homologous recombination deficiency, MSI/MMR status, NTRK fusions and KRAS alleles; third, single-sample subtype assignment and estimation of tumor purity/stromal content; and fourth, orthogonal confirmation of pathway activity by phospho-protein, immune, stromal or metabolic readouts when a pathway-directed trial is considered. This workflow remains investigational, but it makes explicit the information required before stage-aware biology can be translated into patient allocation.

### Genomic stability and hierarchical signaling reorganization

Although PDAC progression is clinically dramatic, the underlying genomic architecture remains relatively stable across stages. Canonical driver alterations in KRAS, TP53, CDKN2A, and SMAD4 are typically established early in tumorigenesis and are not stage-restricted events. This genomic continuity contrasts sharply with the pronounced transcriptomic divergence observed across progression. The biological differences between operable tumors and metastatic lesions therefore arise primarily from hierarchical reorganization of signaling output downstream of shared genomic drivers. KRAS, for example, maintains centrality across stages; however, its downstream signaling balance shifts from MAPK dominance in early disease toward increased PI3K-AKT-mTOR engagement in advanced states. Similarly, MYC activity expands transcriptional amplification capacity without requiring new genomic amplification events in all cases. This indicates that progression in PDAC is best conceptualized as systems-level adaptive rewiring driven by epigenetic modulation, transcription factor redistribution, metabolic stress, and microenvironmental selection pressures rather than stepwise acquisition of novel oncogenic mutations.

A major mechanistic implication is that stable driver genetics can generate changing signaling outputs through non-genetic regulatory layers. KRAS remains the initiating and central oncogenic event in most PDACs, but its downstream balance can shift through receptor tyrosine kinase feedback, phosphatase activity, inflammatory cytokines, nutrient availability, hypoxia, stromal ligands, epigenetic state and therapy-induced selection. Thus, a tumor may remain KRAS-mutant while progressively increasing PI3K-AKT-mTOR, MYC, YAP/TAZ, SRC/FAK or stress-response outputs. This provides a plausible explanation for why early tumors may appear more MAPK/proliferation oriented, whereas advanced or treated tumors may rely more heavily on survival, metabolic and microenvironmental circuits without acquiring new canonical driver mutations. This interpretation also reconciles stable driver genetics with changing drug sensitivity. In early disease, KRAS output may be more tightly linked to proliferation and replication stress, whereas in advanced or treated disease the same oncogenic background may be buffered by feedback receptor signaling, PI3K-AKT-mTOR survival signaling, autophagy, metabolic rewiring and stromal ligands. Therapeutic resistance should therefore be understood as a systems property rather than a simple consequence of new mutations.

### Non-linear evolution and convergent metastatic adaptation

While clinical staging implies linear progression, transcriptomic modeling suggests a more complex evolutionary landscape. BL/LA tumors exhibit dual pathway engagement, combining proliferative MAPK signaling with emerging survival signaling and metabolic plasticity. This intermediate configuration reflects a state of biological instability and adaptability. Primary tumors from metastatic patients and liver metastases, however, demonstrate partial convergence toward survival-dominant and metabolically specialized states. Rather than representing simple quantitative escalation, metastatic competence appears to arise from selective amplification of efficient survival programs and suppression of nonessential pathways. The marked Euclidean separation between operable tumors and liver metastases supports a model of adaptive specialization. Liver metastases are not merely later versions of primary tumors; they represent metabolically refined ecosystems shaped by the hepatic microenvironment, oxygen gradients, lipid availability, and immune tolerance. This suggests that PDAC evolution involves both progressive refinement and convergent adaptation toward energetically efficient metastatic phenotypes.

### Stage-specific mechanisms of therapeutic resistance

Adaptive resistance in PDAC frequently involves kinase phosphorylation, ubiquitin-dependent proteostasis, alternative splicing, RNA methylation, chromatin remodeling and feedback reactivation of signaling pathways. MEK or EGFR-RAS pathway inhibition can be bypassed by ERK rebound, RTK upregulation, PI3K-AKT activation or YAP/TAZ-dependent transcriptional compensation. Similarly, cytotoxic and metabolic stress can select for enhanced autophagy, unfolded-protein response, NRF2-mediated antioxidant capacity, mitochondrial remodeling and proteasome dependence. These mechanisms may explain why transcriptomic pathway enrichment identifies a vulnerability but does not necessarily predict single-agent efficacy. Post-transcriptional regulation is particularly relevant in PDAC progression. For example, aberrant splicing programs such as the CLK1/SRSF axis can modify METTL and Cyclin L2 isoform usage and promote pancreatic cancer growth and metastasis, illustrating how signaling adaptation can occur downstream of stable genomic drivers. In addition, chemotherapy resistance can be shaped by non-genetic targetable states; the recently proposed sulindac-derived K-80,003 strategy with nab-paclitaxel/gemcitabine highlights how drug-resistant PDAC may depend on adaptive biological programs not captured by mutation status alone. In future validation studies, RNA-based pathway calls should therefore be paired with phosphoproteomics to assess signaling flux, proteomics to detect pathway effector abundance, chromatin or methylation profiling to capture epigenetic state, and spatial methods to distinguish tumor-cell-intrinsic programs from CAF, macrophage, endothelial or immune-cell contributions. This multi-layer validation is particularly important for adaptive resistance, where the decisive event may be pathway reactivation rather than transcriptional induction.

The stage-resolved pathway architecture provides a mechanistic framework for understanding therapy resistance. In operable disease, resistance to cytotoxic chemotherapy may emerge through compensatory activation of PI3K-AKT-mTOR survival signaling following replication stress. BL/LA tumors possess metabolic plasticity that allows dynamic switching between glycolytic and oxidative states, potentially conferring resistance to monometabolic targeting strategies. Primary metastatic tumors exhibit reinforced mitochondrial dependency and MYC-driven transcriptional amplification, enabling sustained biosynthetic output under therapeutic pressure. This may reduce sensitivity to single-pathway inhibition and necessitate combinatorial targeting of survival and metabolic circuits. Liver metastases display enhanced redox buffering (NRF2 axis), proteostasis activation (XBP1, ATF4, unfolded protein response), and immune exclusion, collectively generating a highly resilient phenotype. These features likely contribute to resistance against both cytotoxic and immunotherapeutic approaches. Thus, therapeutic escalation without biological stratification may inadvertently select for progressively stress-adapted and treatment-resistant states.

### Limitations

Several limitations warrant consideration. Transcriptomic analyses do not directly measure protein activity or post-translational modifications, which are critical determinants of pathway function. Bulk profiling may obscure intratumoral heterogeneity and stromal contributions. Additionally, cross-sectional datasets cannot fully capture dynamic adaptation under therapeutic pressure. Functional validation in organoid systems, patient-derived xenografts, and longitudinal sampling frameworks will be essential to confirm inferred stage-specific vulnerabilities.

Additional limitations have been clarified. First, cross-sectional datasets cannot distinguish true temporal evolution from selection bias introduced by resectability, metastatic tropism, sampling site, treatment history or tissue quality. Second, bulk transcriptomes merge tumor, stromal and immune signals, and changes attributed to tumor-cell rewiring may partially reflect altered cellular composition. Third, most proposed biomarkers lack validated thresholds, clinical-grade assays and prospective evidence. Fourth, several biologically rational therapeutic strategies in PDAC have failed clinically, underscoring the need to separate mechanistic plausibility from translational readiness. These limitations support a cautious interpretation of the framework as a platform for hypothesis generation and trial design. A further limitation is that the proposed model does not yet define clinically validated cutoffs for pathway positivity. Until such thresholds are prospectively established, stage-aware signatures should be used to enrich trial hypotheses and select correlative endpoints, not to exclude patients from standard treatments or to assign off-label targeted therapy.

### Future directions

Future investigations should integrate single-cell transcriptomics, spatial profiling, and phosphoproteomics to resolve cell-type-specific and microenvironmental contributions to stage-dependent rewiring. Longitudinal sampling during therapy could reveal adaptive trajectory shifts and resistance evolution. Ultimately, PDAC management may require dynamic, stage-aware therapeutic algorithms that incorporate real-time pathway activation profiling. Such an approach recognizes progression not simply as tumor growth but as structured biological evolution. Future trials should ideally include prospective subtype assignment, pre-specified pathway biomarkers, treatment-line stratification, metastatic-site annotation and pharmacodynamic sampling. Adaptive designs may be particularly suitable, allowing patients to be assigned not only by stage but also by tumor-intrinsic subtype, stromal/immune state and evidence-supported pathway activation. This would test whether stage-aware biology improves patient selection beyond current anatomical staging and established molecular classifiers. A useful next step would be a prospective observational-translational study in which resectable, BL/LA, metastatic-primary and metastatic-lesion samples are profiled using the same genomic, transcriptomic, spatial and phosphoproteomic workflow. Such a study would test whether the proposed trajectory remains valid after controlling for subtype, purity, treatment exposure and metastatic site, and would provide the biomarker thresholds needed for subsequent interventional trials.

## Funding

This research received no specific grant from any funding agency in the public, commercial, or not-for-profit sectors.

## CRediT authorship contribution statement

**Eduardo Chuluyan:** Writing – original draft, Conceptualization. **Daniel Grasso:** Writing – original draft, Conceptualization. **Analia Pasqua:** Writing – original draft. **Carla Remolins:** Writing – original draft. **Andrea Paes de Lima:** Writing – original draft. **Kevin Matamoros:** Writing – original draft. **Diego Guerrieri:** Writing – original draft. **Gustavo Kohan:** Writing – original draft. **Florencia Gottardo:** Writing – review & editing. **Martin Ledesma:** Writing – review & editing. **Juan Garona:** Writing – review & editing. **Carlos Davio:** Writing – original draft. **Agustin Yaneff:** Writing – original draft. **Maria Noe Garcia:** Writing – original draft. **Daniela Papademetrio:** Writing – original draft. **Maria Eugenia Pasqualini:** Writing – original draft. **Luis Sarotto:** Writing – original draft. **Jonathan Garnier:** Writing – original draft. **Brice Chanez:** Writing – original draft. **Oscar Mazza:** Writing – original draft. **Nicolas Fraunhoffer:** Writing – original draft. **Nelson Dusetti:** Writing – original draft. **Juan Iovanna:** Writing – original draft, Data curation, Conceptualization.

## Declaration of competing interest

The authors declare that they have no competing financial or non-financial interests, nor any personal or professional relationships, that could be construed as influencing the results or interpretation of this work.
